# WHO Thematic Platform for Health Emergency and Disaster Risk Management Research Network (TPRN): Report of the Kobe Expert Meeting

**DOI:** 10.3390/ijerph16071232

**Published:** 2019-04-06

**Authors:** Ryoma Kayano, Emily YY Chan, Virginia Murray, Jonathan Abrahams, Sarah Louise Barber

**Affiliations:** 1 World Health Organization Centre for Health Development, Kobe 651-0073, Japan; barbers@who.int; 2 Collaborating Centre for Oxford University and Chinese University of Hong Kong for Disaster and Medical Humanitarian Response (CCOUC), School of Public Health and Primary Care, The Chinese University of Hong Kong, HongKong, China; Emily.chan@cuhk.edu.hk; 3 Public Health England, London SE1 8UG, UK; Virginia.Murray@phe.gov.uk; 4 Country Health Emergency Preparedness and International Regulations Department, WHO Health Emergencies Programme, World Health Organization, CH-1211 Geneva, Switzerland; abrahamsj@who.int

**Keywords:** health emergency and disaster risk management (Health EDRM), Sendai Framework for Disaster Risk Reduction 2015–2030, WHO Thematic Platform for Health EDRM, health data, psychosocial, risk communication, capacity building, research methods, ethics

## Abstract

The WHO Thematic Platform for Health Emergency and Disaster Risk Management Research Network (TPRN) was established in 2016 in response to the Sendai Framework for Disaster Risk Reduction 2015–2030. The TPRN facilitates global collaborative action for improving the scientific evidence base in health emergency and disaster risk management (Health EDRM). In 2018, the WHO convened a meeting to identify key research questions, bringing together leading experts from WHO, TPRN, World Association for Disaster and Emergency Medicine (WADEM), and the Japan International Cooperation Agency, and delegates to the Asia Pacific Conference on Disaster Medicine (APCDM). The meeting identified research questions in five major areas for Health EDRM: health data management, psychosocial management, community risk management, health workforce development, and research methods and ethics. Funding these key research questions is essential to accelerate evidence-based actions during emergencies and disasters.

## 1. Introduction

Over the past few decades, the frequency of disasters has increased as a result of a number of risk drivers including unplanned urbanization and unmitigated climate change. The impact of many of these disasters on human health has also become more severe, due in part to increasing numbers of vulnerable populations, including older persons. The 2015 Third UN World Conference on Disaster Risk Reduction (WCDRR) established the Sendai Framework on Disaster Risk Reduction 2015–2030 (SFDRR), which introduced a framework for action across the disaster risk management (DRM) continuum (prevention, preparedness, response, and recovery) and sought to enhance the resilience of communities and health and social systems. With more than 30 references to health issues specifically, the framework includes health in its goal as “the substantial reduction of disaster risk and losses in lives, livelihoods and health ….” [1]. It also emphasizes the importance of improving the scientific evidence base to advance health emergency and disaster risk management (Health EDRM).

In response, World Health Organization (WHO) established the WHO Thematic Platform for Health EDRM Research Network (TPRN) to promote global collaboration among academia [2,3], government officials and other stakeholders to generate better scientific evidence to inform policy and practice for managing health risks associated with emergencies and disaster. In 2017, leaders of this research network published a review paper on the Sendai Framework implementation and recommendations on Health EDRM research [4,5]. The paper highlighted the critical importance of research before, during and after disasters (not only the acute phase) and gave consideration to the following: a holistic approach, including physical, mental and psychosocial health and well-being; identifying populations at risk with specific health needs; standardization of needs assessments, standardization of evaluation methodologies and reporting systems for countries, communities and individual cases; multidisciplinary and multi-sectoral approaches; and a review of research for informing better policy development and implementation.

To accelerate research in Health EDRM, the WHO organized a meeting to identify key research gaps and questions by convening the leading experts from WHO, World Association for Disaster and Emergency Medicine (WADEM), and Japan International Cooperation Agency (JICA), and delegates to the Asia Pacific Conference on Disaster Medicine (APCDM). The WHO Kobe Center (WKC) organized the meeting as one of the programs during APCDM 2018 on 17 October 2018, in Kobe, Japan [6].

## 2. Materials and Methods

The purpose of the meeting was to identify concrete research questions based on the established overarching priorities in line with the published reviews of research needs conducted by the coordinators of the WHO Thematic Platform for Health EDRM Research Network (TPRN) [4,5]. The selection of the research themes, meeting participants and research questions were conducted as below.

As a first step, an open-ended consultation was conducted with the ten TPRN coordinators. Background information from the above-mentioned references [4,5] was shared for the consultation. Through this process, the first draft of the expert meeting agenda with potential discussion themes and background information was developed. Based on the potential discussion themes, 25 participants from 10 countries for the expert meeting were selected based on their global expertise in responding to health emergencies and natural disasters in consultation with the TPRN coordinators and APCDM organizers. They included the WHO Health-EDRM responsible officers in headquarters and regional offices in the Asia Pacific Region (Western Pacific Region and Southeast Asia Region), the TPRN co-chairs and representatives from international, regional or national organizations and societies involved in Health-EDRM (Public Health England, the Chinese University of Hong Kong, WADEM, Association of South-East Asian Nations (ASEAN) Secretariat, JICA, Government of Thailand, Japan Disaster Medical Assistance Team Secretariat; and leading researchers selected based on their previous academic works and Health-EDRM related activities.

As a second step, another consultation to finalize the major research themes was conducted with all of the potential 25 participants to obtain their feedback, and five major discussion themes were selected (Table 1). Based on the final confirmed meeting themes, participants were then selected to cover all the five research themes through consultation with stakeholders. Through this process, 32 experts from 12 countries (Australia, Canada, Hong Kong, India, Indonesia, Japan, Myanmar, New Zealand, Philippines, Thailand, UK, USA) were selected for the expert meeting. The meeting agenda with the five major discussion themes and their background information was shared with all the participants.

As a third step, the expert meeting was conducted as a session of APCDM. Two rounds of group discussions were conducted. The discussion on each theme was facilitated by a lead discussant selected based on their expertise and previous research under each area in Health-EDRM (Table 1). After the group discussions, a plenary discussion was conducted to integrate the results of the group discussions and reach consensus among the participants.

## 3. Key Research Gaps and Questions in Five Key Research Areas

The process resulted in a series of questions listed in Table 1.

The first research area was health data management before, during and after emergencies and disasters. Experts highlighted the significant progress made through the development of the WHO Emergency Medical Team (EMT) Minimum Data Set (MDS) in 2017 [7], enabling standardized data collection and reporting by EMTs dispatched to emergency areas. However, it was noted that implementation was not optimal. Further research is needed on reducing the implementation barriers in national and regional contexts, and the supplementary data required for broader public health action.

The second area was psychosocial management. Experts identified research priorities in the classification of mental health and psychosocial risk, standardization of methods for screening, diagnosis and treatment, and the identification of characteristics of risk and resilience among affected individuals and communities.

The third research area was community risk management and risk literacy. Experts identified the need to strengthen the components of research architecture to support research for Health EDRM, the means to translate research to policy and practice, and research to advance the technology of information and data management and communication for risk communication for Health EDRM, with the focus on an effective and localized risk communication approach to meet the requirements of the local community.

The fourth area was health workforce development. Experts identified knowledge gaps in a common understanding of relevant knowledge and competencies required for the Health EDRM workforce as well as the contents for in-house training/professional development and their interaction with stakeholders, and in understanding how to sustain the development of the local health workforce for Health EDRM, foster positive interactions between external support workers and the local workforce and the effective transition to recovery, integrating measures to reduce risks of future events and build stronger systems. More understanding is needed about how countries can strengthen Health EDRM through disaster risk management training programs, and how they are able to retain, motivate and utilize trained personnel, including for deployment.

The fifth area was research methods and ethics. To move beyond case studies, key research priorities including the basic definitions of research methods and technical terms in the field of Health EDRM setting, standardizing impact evaluation methodologies, and promoting publication of result in a systematic way. Lastly, the group identified the need for best practices in addressing national health systems, and cultural and religious issues. A research methods resource and book were thought to be important additions to the TPRN tools.

## 4. Follow-Up Actions

The expert meeting focused on identifying the key research needs in five major areas through formulating research questions. Specific approaches in responding to each research question should be discussed and implemented with additional studies and literature reviews conducted in a systematic way. These include: (a) conducting more implementation research using currently available health data collection tools (Area 1); (b) standardization of monitoring of long-term psychological consequences, and validation of individual-level and community-level interventions for better psychological consequences (Area 2); (c) identifying the existing challenges for better translation of research findings to policy and programs (Area 3); (d) conducting an inventory of the existing workforce development programs and identifying essential components of training programs (Area 4); and (e) integrating existing knowledge about research methods for Health-EDRM by developing guidance by global experts (Area 5). These follow-up actions are addressed in greater detail in the later theme-based articles.

Given this background, the meeting concluded with the commitment to organize an annual TPRN meeting and utilize other forums in collaboration with partner organizations to promote and update knowledge and experience in Health EDRM research. The meetings will update progress in the key research areas, and further expert consultations will be held to identify further research gaps and possible sources of funding. In 2019, the WKC will function as the secretariat of TPRN in collaboration with WHO HQ. The WHO will establish an information sharing platform to facilitate interactions among TPRN participants about Health EDRM research and science, as well as opportunities for funding, conferences and collaborative research.

## 5. Conclusions

The expert meeting contributed to setting forth some key research gaps to be addressed for the advancement of Health EDRM research. Successful implementation will require further research collaborations through the TPRN initiative, partnerships and resource mobilization.

The expert meeting did not reach consensus through blind rating, voting or a structured questionnaire-based approach, which are recommended by Delphi Method. This limitation will be addressed during the next follow-up expert meeting.

## Figures and Tables

**Table 1:** Key research questions in five major areas.

Area	Research Questions
Area 1 Health data management before, during and after emergencies and disasters Lead discussant: Tatsuhiko Kubo, University of Occupational and Environmental Health, Japan	(a) What are the national and regional challenges inhibiting implementation of the WHO standardized medical data collection systems after emergencies and disasters? (b) What is the broader health-related data needed to inform effective Health emergency and disaster risk management (EDRM), i.e., community vulnerabilities, hospital functional status, infrastructure, lifelines and health workforce?
Area 2 Psychosocial management before, during and after emergencies and disasters, and other medium and long-term effects on the public health and health system Lead discussant: Yoshiharu Kim, National Center for Neurology and Psychiatry, Japan	(a) How can mental health and psychosocial risk be classified using longitudinal and multi-centric studies? (b) How can methods for screening, diagnosis and treatment for affected people be standardized across different settings? (c) How can assets associated with greater community resilience be identified before, during, and after disaster?
Area 3 Community emergency and disaster risk management, including risk literacy and addressing needs of sub-populations Lead discussant: Emily Y.Y. Chan, The Chinese University of Hong Kong	(a) What architecture is needed to support research in Health EDRM including consensus among disciplines and ethics? (b) How can research be better translated to policy and practice across different backgrounds and contexts? (c) What kind of technology for information and data management and communication is needed for risk communication, emergency response and research design?
Area 4 Health workforce development for health emergency and disaster risk management Lead discussant: Jonathan Abrahams, World Health Organization	(a) How can different countries strengthen Health EDRM through disaster risk management training programs, and what strategies will support retention, motivation and deployment of trained people? (b) What are the best practices for sustaining the development of the local health workforce for Health EDRM, fostering positive interactions between external support workers and the local workforce, and enabling the transition to recovery and post-event Health EDRM? (c) What is the common knowledge or competencies required for Health EDRM?
Area 5 Research methods and ethics Lead discussant: Virginia Murray, Public Health England	(a) What are the definitions of research methods and technical terms for Health EDRM? (b) How can impact evaluation methods for intervention and qualitative–quantitative mixed methods be standardized? (c) How can the publication process for Health EDRM research become more systematic and effective? (d) What are the challenges and best practices in addressing national health system, cultural and religious issues before, during and after interventions?
